# Laryngeal mask airway or high-flow nasal cannula *versus* nasal cannula for advanced bronchoscopy: a randomised controlled trial

**DOI:** 10.1183/23120541.00421-2024

**Published:** 2025-02-10

**Authors:** Regina Pikman Gavriely, Ophir Freund, Boaz Tiran, Tal Moshe Perluk, Eyal Kleinhendler, Idit Matot, Amir Bar-Shai, Evgeni Gershman

**Affiliations:** 1 Department of Anesthesiology, Intensive Care and Pain, Tel Aviv Sourasky Medical Center, Tel Aviv, Israel; 2 Faculty of Medicine, Tel Aviv University, Tel Aviv, Israel; 3 Institute of Pulmonary Medicine, Tel Aviv Sourasky Medical Center, Tel Aviv, Israel

## Abstract

**Background:**

Advanced bronchoscopic procedures have become a widely prevalent evaluation and treatment modality. These procedures require appropriate sedation and respiratory support. This study directly compares three respiratory support methods during advanced bronchoscopy.

**Methods:**

This three-arm, prospective, block randomised trial included 60 consenting adult patients that were referred for advanced bronchoscopy involving endobronchial ultrasound (EBUS) with transbronchial needle aspiration and met inclusion/exclusion criteria. Patients were randomised to undergo bronchoscopy through a laryngeal mask airway (LMA) or with a high-flow nasal cannula (HFNC) or low-flow nasal cannula (NC), with bronchoscopy performed through a bite block. Demographic, procedural and clinical parameters were compared between the three groups, including complications, oxygenation, ventilation and need for intervention.

**Results:**

Analysis according to intention to treat was made for the 20 patients in each arm. There were no significant differences in demographic parameters, pre-morbidities and procedure type and duration between groups. Hypoxia was significantly more common in the NC group (90%) compared with the LMA (45%) and HFNC (26%) groups (p<0.01). The need for interventions and their number were also lower in the LMA (40%) and HFNC (52.6%) groups compared with the NC group (90%, p<0.01). A multivariate analysis confirmed both HFNC and LMA as independent predictors of a lower rate of recurrent desaturation events and fewer complications in general compared with NC.

**Conclusion:**

In this prospective randomised trial, we demonstrated the advantages of using LMA or HFNC over NC during advanced bronchoscopy with EBUS.

## Shareable abstract

**This randomised controlled trial showed that use of LMA or HFNC during advanced brochoscopic procedures had equivalent performance, and both were superior to nasal cannula in terms of oxygenation, ventilation and need for respiratory support intervention** https://bit.ly/4d9PAXc

## Introduction

In the past two decades, endobronchial ultrasound (EBUS) with transbronchial needle aspiration (TBNA) and other advanced bronchoscopic procedures have become major diagnostic tools for benign and malignant pulmonary and mediastinal findings [1, 2]. Their use often results in prolonged examinations, which may require deeper sedation in comparison to regular bronchoscopic examinations for a safe and efficient test [3]. The EBUS probe has a wider diameter and involves prolonged uninterrupted attachment to the airway wall, causing airway irritation, cough, discomfort and autonomic responses. To ameliorate these responses, a proper level of local anaesthesia and sedation are required [4], sometimes to the point of general anaesthesia [5]. Side effects of sedation may include desaturation, hypoventilation and haemodynamic complications [4]. In addition, many of the patients undergoing these invasive procedures suffer from chronic lung illnesses and obesity, increasing the risk of hypoxic respiratory complications during the examination [6, 7]. Therefore, there is a need to preserve proper oxygenation and respiratory support during those procedures [1].

Various medical centres have different sedation and oxygenation protocols according to the specific characteristics of the patient and the procedure [8–11]. Although most studies did not find major differences between sedation protocols, deeper sedation was necessary for longer and more complex procedures [12].

As to the method of oxygenation and ventilation, a spectrum of options varying from spontaneous breathing with supplemental oxygen *via* nasal cannula to endotracheal intubation and positive pressure ventilation are available [13, 14]. Some centres use laryngeal mask airway (LMA) as a comfortable method for securing the airway, performing the endoscopic examination and ventilating the patient if necessary [15, 16]. Recently, there is an increasing interest in high-flow nasal cannula (HFNC) in advanced bronchoscopic examinations, including EBUS [17–19]. HFNC delivers actively heated, humidified medical gas *via* air/oxygen blender with varying flow and inspiratory oxygen fraction (*F*_IO_2__). In addition to providing constant *F*_IO_2__, HFNC enhances CO_2_ clearance and reduces the work of breathing [20].

In this study, our goal was to compare the safety and efficacy of respiratory support during advanced bronchoscopy using either LMA or HFNC compared with using a standard nasal cannula.

## Methods

### Study design

This is a single-centre, three-arm, randomised, prospective study of patients who underwent advanced bronchoscopy in a large tertiary centre between 1 October 2022 and 31 October 2023. The local ethics committee (IRB TLV-0468-22) approved the study protocol and it was planned and conducted in accordance with the ethical guidelines set forth in the Declaration of Helsinki. All participants signed informed consent after receiving details about the study from the research team.

### Study population and procedure

Subjects that were planned to undergo ambulatory EBUS and advanced bronchoscopy were included if they met the following criteria: 1) bronchoscopy examination with EBUS and TBNA with or without additional bronchoscopic procedures; 2) medical condition that allows the patients to undergo sedation and examination using any of the three interventions (haemodynamic and respiratory stability); 3) having oxygen saturation >92% in room air at baseline; and 4) consented to participate in the study. Patients with any of the following were excluded: 1) age under 18 years; 2) inability to sign informed consent; and 3) pregnancy. Following informed consent, patients were randomised to one of three interventions: NC, HFNC or LMA. Patients were blinded to the method of oxygenation during their exam. Demographic and clinical data were collected during the patient pre-procedural visit and a predefined database was filled out accordingly.

Vital signs were collected during the procedure (at baseline, at intervals of 3 min during the procedure, and at intervals of 5 min in the 30 min after the procedure (recovery)) and included heart rate, oxygen saturation (initially at room air and later on oxygen supply per intervention), blood pressure and a percutaneous CO_2_ (pctCO_2_) (Sentec) patient monitor, with its probe on the frontal bone on the left side, an acceptable CO_2_ measurement in the absence of end-tidal CO_2_ [6, 21–23].

All bronchoscopies were performed by experienced pulmonologists and sedation for all procedures was performed and monitored by a dedicated anaesthesiologist, who also enrolled the patients. All bronchoscopies were performed according to a similar sedation and procedural protocol, in accordance with accepted guidelines as previously published [24–26]. In general, patients received local oral anaesthesia using Xylocaine spray (three puffs) and sedation, provided by an anaesthesiologist, with a combination of fentanyl and midazolam given initially, and an addition of propofol in appropriate dosing to reach moderate–deep sedation (RAMSAY score 5–6) as assessed by the anaesthesiologist.

At first, all patients were oxygenated with 4 L·min^−1^ NC for 10 min before anaesthesia commenced, upon achieving the desired sedation level they were randomised (by block randomisation) to receive one of the following oxygenation options:

1) LMA (Ambu Aura40, size 4, connected to Ambu bag with reservoir and 10 L·min^−1^ O_2_)
2) HFNC (100% O_2_, 45 L·min^−1^, Airvo, Bepex)
3) NC (4 L·min^−1^, Unomedical, ConvaTec)

As part of the study protocol, a conversion in between groups was allowed by the discretion of the performing bronchoscopist and anaesthesiologist if found necessary for patient safety and or technical challenges precluding a diagnostic yield.

The bronchoscope (EBUS Olympus) was inserted through the mouth (bite block) in the nasal cannula groups or directly thru the LMA *via* a plastic swivel in the LMA group. Additional local anaesthesia to the trachea and vocal cords was administered using 3 mL Lidocaine 2% solution. During the examination the airways were viewed directly and *via* ultrasonography, samples of suspected masses and paratracheal/perihilar lymph nodes were collected using a 21-G EBUS–TBNA sampling needle. Additional bronchoscopic procedures followed after retrieval of the EBUS using an Olympus bronchoscope (endobronchial biopsy, radial biopsy, cryobiopsy, bronchial brush and bronchoalveolar lavage).

Completion of the procedure was defined by announcement of the pulmonologist and retrieval of the endoscope. Afterwards, subjects were monitored for 30 min in the bronchoscopy suite for vital signs as described earlier. During this time the NC and HFNC groups remained with the NC and HFNC, respectively and those with LMA remained with LMA until awakening and were then oxygenated with NC for the remaining monitoring time.

### Outcome measures

The primary outcomes were respiratory complications: 1) hypoxia, oxygen saturation below 90% for more than 30 s and recurrent hypoxia (more than one desaturation event during the procedure); 2) hypoventilation defined as pctCO_2_ elevation of more than 20 mmHg from baseline, CO_2_ level of 60 mmHg or above and peak CO_2_ value and the time of CO_2_ recovery to baseline value; and 3) change in ventilatory support method, airway manoeuvres such as chin lift and jaw thrust or oxygen enrichment.

Secondary outcomes included airway bleeding (requiring any intervention), systolic blood pressure below 90 mmHg in at least one measurement, need for an intervention to help maintain cardiovascular stability, including the administration of medication to elevate blood pressure, and satisfaction score of patient (1–4 score), operator and anaesthesiologist (measured using 0–10 score after each examination).

### Data analysis

Randomisation was performed in blocks of 10 (6 blocks overall), with the intervention of the first participant in each block chosen by randomly selecting an envelope with one of the interventions written (LMA, NC and HFNC). For sample size calculation, we assumed (based on prior research) a 65% desaturation rate in subjects treated with NC [27]. For an α of 5%, a power of 80% and a minimal detectable effect of 30% decrease in desaturation events in the LMA and HFNC groups, a sample size of 17 subjects in each group is needed (overall 51). We included 20 subjects in each group to account for a 15% incompletion rate.

Variables are presented as absolute numbers and percentages, mean±sd for continuous data if normally distributed and median and interquartile range if not normally distributed. Normal distribution was assessed by Kolmogorov–Smirnov tests. Comparison between the three groups was performed using chi-squared for dual variables, ANOVA for parametric variables and a Kruskal–Wallis test for nonparametric variables. Analyses were made according to the original group allocation (intention-to-treat analysis). Multivariate logistic regression models were conducted to examine independent predictors for the primary outcomes. A p-value <0.05 was considered to be significant. SPSS version 28.0 was used for statistical analyses.

## Results

**FIGURE 1:**
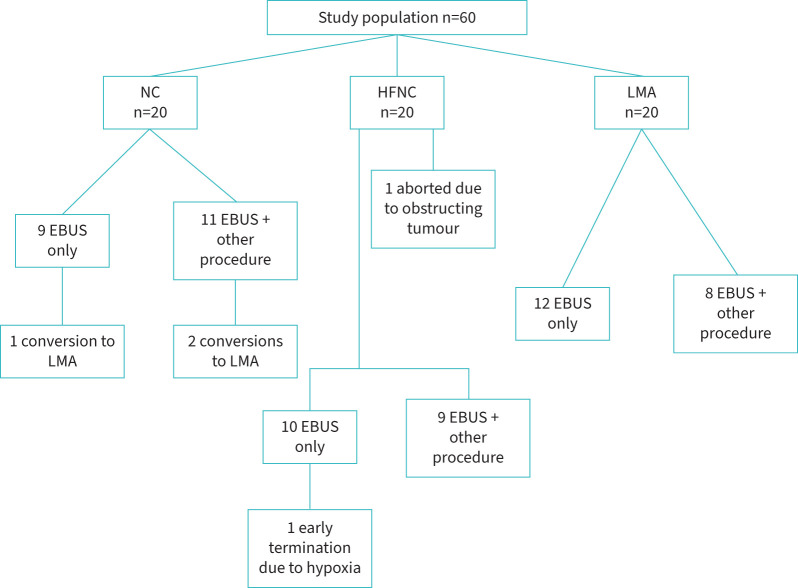
Study population, division into study groups and procedures performed during endobronchial ultrasound (EBUS) examination. NC: low-flow nasal cannula; HFNC: high-flow nasal cannula; LMA: laryngeal mask airway.

From October 2022 to October 2023, 60 patients were recruited for the study (mean age of 65.45±12 years, 63% male), with 20 in each arm. Figure 1 outlines the enrolment flow chart. All examinations involved EBUS–TBNA and in 46.67% (n=28), an additional procedure was performed, in a similar rate between the groups (p=0.64). Additional procedures included endobronchial biopsy, radial biopsy, cryobiopsy, bronchial brush and bronchoalveolar lavage (a full list of procedures in each group is provided in supplementary table 1).

Clinical and demographic characteristics of the study groups are presented in table 1. The three groups were comparable regarding demographic and clinical parameters. Body mass index (BMI) was higher in the LMA group compared with the HFNC group (p=0.01). Baseline vital signs were also similar between the groups.

**TABLE 1:** Cohort characteristics and comparison between study groups

	LMA	HFNC	NC	P1	P2	P3
**Subjects, n**	20	20	20			
**Age, years**	67.4±11.1	63.5±14.8	65.5±10.8	0.65	0.82	0.77
**Male**	13 (65)	15 (75)	10 (50)	0.33	0.26	0.06
**BMI, kg·m^−2^**	28.5±4.4	25.5±4.5	25.0±3.6	0.01	0.21	0.81
**Smoker**	16 (80)	17 (85)	14 (70)	0.77	0.72	0.52
**Pack-years**	40 (22.5–75)	60 (47.5–72.5)	50 (20–60)	0.23	0.81	0.51
**COPD**	5 (25)	9 (45)	7(35)	0.26	0.49	0.65
**ASA score**	2.5±0.6	2.65±0.8	2.55±0.5	0.53	0.70	0.75
**Mallampati**	2.55±0.8	2.05±0.9	2.55±0.8	0.10	1.00	0.10
**Initial vital signs**						
SBP, mmHg	151.3±20	140.8±29	145.9±23	0.13	0.51	0.41
DBP, mmHg	85.1±10.9	84.8±16.7	82.8±11.6	0.55	0.41	0.95
Pulse	74.05±14.2	72.8 ±17.9	78.15±20.3	0.77	0.58	0.48
Saturation	99.5±1.8	98.4±1.8	99.3±1.8	0.08	0.58	0.09
pctCO_2_^#^	40.4±6.2	43.6±5.7	40.6±3.7	0.07	0.89	0.05

Data are presented as n (%), mean±sd or median (interquartile range). LMA: laryngeal mask airway; HFNC: high-flow nasal cannula; NC: low-flow nasal cannula; P1: comparison between LMA *versus* HFNC; P2: comparison between LMA and NC; P3: comparison between HFNC and NC; BMI: body mass index; ASA: American Society of Anesthesiologists; SBP: systolic blood pressure; DBP: diastolic blood pressure; pctCO_2_: percutaneous CO_2._^#^: Data for pctCO_2_ measurements were collected and analysed from 19 patients in the LMA group, 17 patients in the HFNC group and 19 patients in the NC group, due to percutaneous CO_2_ measurement device malfunction.

Procedural characteristics of the study groups are presented in table 2. The LMA group had used a lower dosage of midazolam compared with the HFNC group (mean 1.3±0.5 mg *versus* 1.9±0.6 mg; p<0.01). This group had also received a lower fentanyl dosage compared with the NC group, although it was not statistically significant (mean 82.5±24.5 μg *versus* 98.8±5.6 μg; p=0.09). Other procedure related parameters were comparable between the three groups.

**TABLE 2:** Procedural characteristics and comparison between the study groups

	LMA	HFNC^#^	NC	P1	P2	P3
**Subjects, n**	20	19	20			
**Duration, min**	33.6±10.5	38.1±14.4	42.6±17.3	0.37	0.10	0.44
**Propofol, mg**	278±149.4	331.6±165.4	245.5±118.1	0.19	0.62	0.06
**Fentanyl, μg**	82.5±24.5	94.7±15.8	98.8±5.6	0.19	0.09	0.75
**Midazolam, mg**	1.3±0.5	1.9±0.6	1.7±0.7	<0.01	0.08	0.37
**Station number**	2 (1–3)	3 (2–3)	2 (1–3)	0.06	0.45	0.22
**Biopsy samples**	4 (2–5.5)	5 (3–6)	4.5 (2.5–6)	0.12	0.64	0.33

Data are presented as n, mean±sd or median (interquartile range). LMA: laryngeal mask airway; HFNC: high-flow nasal cannula; NC: low-flow nasal cannula; P1: comparison between LMA *versus* HFNC; P2: comparison between LMA and NC; P3: comparison between HFNC and NC. ^#^: Data were collected and analysed from 19 patients in the HFNC group. One procedure was terminated and no endobronchial ultrasound was performed due to bronchoscopic findings at the beginning of the procedure (obstructing tumour).

### Primary outcomes

Primary and secondary study outcomes are presented in table 3. Complications were more common in the NC group (90%) compared with the LMA (55%; p=0.01) and HFNC (47%; p<0.01) groups. The rate of hypoxaemic complications (any desaturation and recurrent desaturation) was higher in the NC group compared with the LMA and HFNC groups (p<0.01).

**TABLE 3:** Primary and secondary outcomes

	LMA	HFNC^#^	NC	P1	P2	P3
**Subjects, n**	20	19	20			
**Primary outcomes**						
Any complications	11 (55)	9 (47)	18 (90)	0.63	0.01	<0.01
Desaturation						
Any desaturation	9 (45)	5 (26)	18 (90)	0.22	<0.01	<0.01
Recurrent desaturation	1 (5)	1 (5)	15 (75)	0.97	<0.01	<0.01
Desaturation events	0 (0–1)	0 (0–1)	4 (2–6.5)	0.35	<0.01	<0.01
Hypoventilation, pctCO_2_^¶^						
pctCO_2_ increase, mmHg	16.9±10.1	21.5±9.1	19.3±7.1	0.14	0.22	0.50
pctCO_2_ increase >20 mmHg	6 (31.6)	9 (56.25)	9 (47.37)	0.14	0.32	0.60
Maximal pctCO_2_, mmHg	57.3±10.5	65.6±10.3	60.1±5.8	0.02	0.11	0.05
Maximal pctCO_2_ >60 mmHg	7 (37)	11 (70)	10 (50)	0.06	0.41	0.26
Time to pctCO_2_ recovery >30 min	12 (63.2)	6 (37.5)	16 (80)	0.23	0.14	0.01
**Intervention, respiratory support**						
Patients requiring intervention	8 (40)	10 (52.6)	18 (90)	0.43	<0.01	0.01
Number of interventions	0 (0–1)	1 (0–1)	2 (1–2)	0.40	<0.01	<0.01
**Secondary outcomes**						
Bleeding (1–4 scale)	0 (0–1)	1 (1–1)	1 (0.5–1.5)	0.05	0.08	0.95
Hypotension, SBP <90 mmHg	4 (20)	1 (5.3)	4 (20)	0.17	1.00	0.17
**Conclusive pathology**	19 (100)	19 (100)	17 (89)	1.00	0.15	0.15
**Satisfaction**						
Operator (0–10 scale)	8.4±1.14	6.8±1.44	6.35±1.84	<0.01	<0.01	0.57
Anaesthesiologist (0–10 scale)	7.75±1.16	7±1.2	5.9±1.37	0.07	<0.01	0.01
Patient (1–4 scale)	3.45±0.69	3.2±0.63	3.3±0.66	0.26	0.48	0.69

Data are presented as n, n (%), mean±sd or median (interquartile range). LMA: laryngeal mask airway; HFNC: high-flow nasal cannula; NC: low-flow nasal cannula; P1: comparison between LMA *versus* HFNC; P2: comparison between LMA and NC; P3: comparison between HFNC and NC; pctCO_2_: percutaneous CO_2_; SBP: systolic blood pressure. ^#^: Data were collected and analysed from 19 patients in the HFNC group. One procedure was terminated and no endobronchial ultrasound was performed due to bronchoscopic findings in the beginning of the procedure (obstructing tumour). ^¶^: Data for pctCO_2_ measurements were collected and analysed from 19 patients in the LMA group, 16 patients in the HFNC group and 19 patients in the NC group, due to pctCO_2_ measurement device malfunction.

The degree of CO_2_ increase from baseline was similar between the LMA, HFNC and NC groups, and was 16.87 mmHg, 21.54 mmHg and 19.25 mmHg, respectively (p=0.284). The peak CO_2_ value was higher in the HFNC group with 65.5 mmHg compared with 57.3 mmHg in the LMA group and 60.07 mmHg in the NC group. Recovery of CO_2_ values to baseline within 30 min after bronchoscopy completion was not reached in 37.5% of patients in the HFNC group, as opposed to 63.2% in the LMA group (p=0.23) and 80% in the NC group (p=0.01).

In the NC group there were more interventions performed, including crossover to a different respiratory support method, chin lift and oxygen enrichment. The NC group also required more interventions in total compared with the HFNC and LMA groups (median of 2 *versus* 1 and 0, respectively; p<0.01).

Three procedures in the NC group had to be converted to LMA at the bronchoscopist decision due to inadequate saturation and airway bleeding. Following conversion to LMA, all three procedures were successfully completed without further complications. One examination in the HFNC group was aborted shortly after its beginning due to an obstructing tumour in the trachea. Another patient from the HFNC group had an early termination of the test due to recurrent hypoxia. There were no serious adverse events in our series, including hospital admissions or death secondary to the procedure.

In multivariate analysis (table 4), by using NC as the reference, LMA (adjusted OR 0.134, 95% CI 0.02–0.80; p=0.02) and HFNC (adjusted OR 0.110, 95% CI 0.02–0.64; p=0.01) were independent predictors for less procedural complications. In a second multivariate analysis (table 5), also adjusted for the time duration of the procedure, both HFNC (AOR 0.007, 95% CI 0.00–0.13; p<0.01) and LMA (AOR 0.009, 95% CI 0.00–0.19; p<0.01) were independent predictors of a lower rate of recurrent desaturation events.

**TABLE 4:** Multivariate analysis of predictors for procedural complications, including desaturation, bleeding and hypotension

Variable	Adjusted OR	95% CI	p-value
**Female sex**	2.031	0.53–7.84	0.30
**BMI**	1.041	0.89–1.22	0.62
**COPD**	2.015	0.52–7.88	0.31
**Nasal cannula**	Ref.		
**High-flow nasal cannula**	0.110	0.02–0.64	0.01
**Laryngeal mask airway**	0.134	0.02–0.80	0.02

BMI: body mass index.

**TABLE 5:** Multivariate analysis of predictors for recurrent (at least two) desaturation events

Variable	Adjusted OR	95% CI	p-value
**Female sex**	0.210	0.02–2.92	0.245
**BMI**	1.046	0.82–1.33	0.714
**COPD**	0.986	0.12–8.44	0.989
**Procedure duration**	1.042	0.097–1.12	0.268
**Nasal cannula**	Ref.		
**High-flow nasal cannula**	0.007	0.00–0.13	<0.01
**Laryngeal mask airway**	0.009	0.00–0.19	<0.01

BMI: body mass index.

### Secondary outcomes

The average degree of airway bleeding was 1 in the HFNC and NC groups and 0 in the LMA group (p=0.05–0.95). Hypotension events occurred in four patients in the LMA group, one in the HFNC group and four in the NC groups without significant difference (p>0.05 for all) (table 3).

Diagnostic yield was 93% on average, ranging from 100% in the LMA and HFNC groups to 89% in the NC group (p>0.05 for all). Operator and anaesthesiologist satisfaction scores were highest in the LMA group when compared with the two other groups (p<0.01 for both). Patient satisfaction (on a scale of 1–4) was high in all of the groups (3.45, 3.2 and 3.3, respectively), without significant differences between the groups (table 3).

## Discussion

Various respiratory support methods for bronchoscopy are utilised by different centres but there is a lack of evidence and direct comparisons on the use of the three main noninvasive oxygenation methods for advanced bronchoscopy: NC, LMA and HFNC. To our knowledge, this is the first randomised controlled study to directly compare three methods of respiratory support during EBUS with advanced bronchoscopy procedures in spontaneously breathing patients. Significantly fewer complications, mainly desaturation events, and less need for intervention were found in the LMA and HFNC groups compared with the NC group.

The findings from the current study are congruent with previous studies comparing HFNC with NC [18, 28–33]. However, there are some differences between those studies and our work. Ucar *et al*. [28] compared the use of HFNC and NC during EBUS. Similar to our findings, they demonstrated significantly less desaturation in the HFNC group when compared with the NC group (6% *versus* 26%, respectively). They, however, used midazolam as a single sedation agent, which may explain their lower rate of desaturations in the NC group (26%) when compared with the results from our study (90%) in which propofol was also administered. In addition, the patients from the Ucar *et al.* study experienced more discomfort in the NC group as opposed to similar results between the groups in our work, which might reflect a shallower level of sedation. In a similar work by Irfan *et al.* [29], a lower rate of desaturation was found in the HFNC group compared with the NC group (5% *versus* 55%, respectively). In their work, Irfan *et al.* did not find any difference in the CO_2_ measurements during or after the procedure.

Darie *et al.* [30] focused on COPD patients, a subpopulation prone to pulmonary tumours and infections who often undergoing bronchoscopy (35% of patients in our work) and with an increased risk of desaturation. They found less desaturation (oxygen saturation <90%) in the HFNC group compared with the NC group (6.3% *versus* 8.6%; p<0.0001). Douglas *et al.* [31] also demonstrated lower rates of desaturation in their study across the different groups (13% in the HFNC group and 33% in the NC group), compared with our experience. They compared HFNC *versus* NC in EBUS and had a shallower level of sedation (MOAA/S=4 *versus* RAMSAY 5–6) and a shorter procedure duration (22.5 min on average *versus* 38 min) compared with our work.

Alon *et al.* [16] compared the use of LMA *versus* NC during bronchoscopy. They demonstrated lower desaturation rates in the LMA *versus* the NC group (37% and 63%, respectively). Alon *et al.* used pctCO_2_ measurements, much like our work, but did not find significant differences in this parameter between the groups. In addition, Alon *et al.* had technically simpler and shorter procedure duration (average length of 20 min); accordingly, they had a lower propofol dosage and lower rate of desaturation than in our work.

In a recent study, Wei *et al.* [34] compared NC *versus* supraglottic jet oxygenation and ventilation (SJOV) during deep sedation for bronchoscopy. SJOV involves positive pressure ventilation through a specialised one-nostril nasal cannula. In their work, Wei *et al.* used a combination of opiates and propofol and reached moderate–deep sedation levels (MOAA/S 1–2), similar to our study. Their study demonstrated desaturation in 86% of the NC group (a similar rate to the 90% desaturation in the NC group found in our study). The SJOV group had significantly fewer desaturations (9.1%) and need for intervention. The lower rate of desaturation in the SJOV group compared with the LMA and HFNC groups in our study may be attributed to positive pressure ventilation *versus* spontaneous ventilation in our study, in addition to longer procedures and a higher dosage of propofol in our work.

In our study, we have found significant differences between the CO_2_ measurements in the different intervention groups. The highest average CO_2_ was found in the HFNC group and the lowest was in the LMA group (p=0.03). Though HFNC improves ventilation and CO_2_ removal [20, 35], there might have been a respiratory drive depression due to high inhaled oxygen levels in the HFNC group, which received 100% *F*_IO_2__. This phenomenon in COPD patients might be the result of either eliminating hypoxic vasoconstriction, depressing respiratory drive or changing the haemoglobin–CO_2_ dissociation curve. Surprisingly, the HFNC group that had the highest average CO_2_ also had the fastest CO_2_ recovery to baseline. This result is consistent with HFNC ability for CO_2_ washout, especially after anaesthesia, with its respiratory depression, wears off. Another reason for accelerated CO_2_ recovery in the HFNC group is the fact that this group had HFNC on during recovery, whereas the other two groups were with NC (after LMA removal). Although not all results were statistically significant, the LMA group showed the lowest increase in CO_2_ and the lowest peak CO_2_ values. This can be attributed to airway resistance and collapse reduction using LMA [36]. The use of LMA is also associated with a lower ratio of dead space to tidal volume, which is comparable to intubation, possibly leading to improved CO_2_ clearance [37, 38].

In terms of operator comfort, the LMA group scored the highest. This might be attributed to the fact that in this group the airway access for bronchoscopy was made *via* the LMA itself. From an aesthetic point of view LMA, and to a lesser degree HFNC, were preferable to NC, most likely due to less complications and need for intervention and crossover. Since, as previously mentioned, the level of sedation was moderate–deep, there were no events of patient discomfort and patient satisfaction was scored equally highly in all groups.

Our research has several limitations. First, this is a single-centre study with a low number of patients, limiting the power for our secondary outcomes analysis and generalisation of the results. Second, the pulmonologists performing the procedure and anaesthesiologist were not blinded to the intervention. Third, intubation was not assessed by our study and should be included in future prospective controlled trials to evaluate its efficacy compared with the methods presented in this work. Finally, we did not excluded patients based on the need for additional procedures other than EBUS–TBNA during the procedure. While their rate was similar between the groups, we could not exclude its effect on the examination outcomes.

In conclusion, the present study suggests that the use of LMA or HFNC is preferable to NC for oxygenation and respiratory support of spontaneously breathing, moderately deeply sedated patients during advanced bronchoscopy with EBUS. Additional multicentred studies in a larger and diverse population are needed to examine the appropriate oxygenation method for various patients and procedures.

## Supplementary material

10.1183/23120541.00421-2024.Supp1 **Please note:** supplementary material is not edited by the Editorial Office, and is uploaded as it has been supplied by the author.
Supplementary material 00421-2024.SUPPLEMENT

## Data Availability

All the analysis results are given within the manuscript. The study was performed prospectively and according to the regulations of the institution review board, such data could not be openly shared. Request for the dataset supporting our results can be made *via* helsinki@tlvmc.gov.il and will be given by the corresponding author after approval.
