# Supplementary material for: Laryngeal mask airway or high-flow nasal cannula versus nasal cannula for advanced bronchoscopy: a randomised controlled trial
Source: ERJ Open Res. 2025 Feb 10;11(1):00421-2024. doi: 10.1183/23120541.00421-2024 (PMC11808932; doi:10.1183/23120541.00421-2024)
Supplement: Supplementary file 1 [file 00421-2024.SUPPLEMENT.pdf]

Supplementary

Laryngeal mask airway or high flow nasal cannula versus nasal cannula for advanced bronchoscopy – a randomized controlled trial

Table S1, Additional procedures performed during bronchoscopy.

Table S1, Additional procedures performed during bronchoscopy.

Procedure	Nasal cannula	HFNC	LMA
**Endobronchial biopsy**	11	10	8
**Radial biopsy**	3	2	7
**Cryobiopsy**	-	-	1
**Bronchial brush**	1	1	2
**Bronchoalveolar lavage**	4	1	-

Abbreviations: HFNC, high-flow nasal cannula; LMA, laryngeal mask airway.

More than one additional procedure was performed on some of the patients
